# Mediating Effect of Compassion Competence on the Relationship between Caring Behaviors and Quality of Nursing Services in South Korea

**DOI:** 10.3390/healthcare10050964

**Published:** 2022-05-23

**Authors:** Hyunjin Lee, Kawoun Seo

**Affiliations:** 1 College of Nursing, Eulji University, Seongnam-si 13135, Korea; hjlee@eulji.ac.kr; 2 Department of Nursing, Joongbu University, Geumsan-gun 32713, Korea

**Keywords:** caring behavior, empathy, nurses, nursing services, service quality

## Abstract

This study aimed to investigate the mediating effect of compassion competence on the relationship between caring behaviors and the quality of nursing services. Participants included nurses working in South Korean hospitals. Data collected from 1 November to 31 December 2020 were analyzed using a *t*-test, analysis of variance (ANOVA), Pearson’s correlation coefficients, and hierarchical multiple regression. We found that caring behavior had a positive correlation with the quality of nursing services and compassion competence. Moreover, there was a positive correlation between the quality of nursing services and compassion competence. Compassion competence had a significant mediating effect on the relationship between caring behaviors and quality of nursing services. This suggests that nurses’ caring behaviors can enable high-quality nursing services influenced by compassion. Therefore, in order to improve the quality of nursing services, repeated and continuous implementation of training programs including education that can improve compassion competence is necessary.

## 1. Introduction

With improvements in people’s standard of living, as well as their awareness of health-related quality of life, maintaining the quality of nursing services in medical institutions becomes increasingly crucial [1,2]. The quality of nursing services is one of the main indicators to evaluate the performance of hospital management and provide guidelines for customer-oriented hospital management; further, it is found to impact hospital reuse intention [3]. To gain a competitive edge in a medical environment, medical institutions have recently started conducting internal assessments of the quality of nursing services and patient satisfaction and are initiating various activities and research to improve them.

Quality of nursing services refers to the quality of nurses’ patient-directed activities. It indicates the consistency of the results achieved, conformity to standards, and the degree of excellence in nursing [4]. The quality of nursing service is an important variable that can hasten patients’ recovery, ensure the maintenance and improvement of their health, as well as foster patient satisfaction and hospital reuse intention [4,5,6]. Therefore, nurses, who spend most of their time with patients, need to appreciate the importance of the quality of nursing services they provide [7]. Along with the interest in and expectations from the quality of nursing services, the concept of care to improve the quality of nursing is also being re-evaluated [8].

Caring behavior refers to the act of caring for the participants of nursing care itself. This is differentiated from the quality of nursing services which prioritizes the satisfaction of patients receiving nursing care, and these aspects [5,6]. Providing care is a key aspect of nursing, and the concept of care has evolved across time and cultures. Currently, care is not simply an activity that addresses one’s needs and those of others to improve their health status or lifestyle. It involves establishing a therapeutic relationship with patients and helping them through their recovery, with care and sincerity [9,10]. The fundamental elements of caring include identifying and meeting the needs of patients and their families, knowing one’s own abilities, and building relationships with patients [11,12]. In other words, nurses provide and express care to patients depending on their beliefs regarding care [13]. Frequent caring behaviors of nurses help patients feel relieved from fearful and anxious situations and satisfied with the care provided; further, these behaviors have a positive effect on patient-nurse relationships [11,14]. These caring behaviors are related to the degree of empathy of nurses [15] and help meet patients’ individualized needs. Ultimately, it may improve patients’ well-being and the quality of care provided to them [10].

Nursing is an act performed through a mutual relationship between the nurse and the patient. Compassion is an important competence for nurses to understand the patients’ situation, accurately perceive their emotions, and encourage them [15]. Nurses’ compassion helps them respond appropriately to patients’ needs, approach the patients’ subjective experience, provide individualized care, and improve the ability to predict patient behavior [16]. Therefore, when a nurse’s empathy is high, the ability to grasp the patients’ emotional state or non-verbal cues increases; thus, nursing can be provided based on a meticulous understanding. Since empathy is an essential element for providing good nursing care [17], the quality of nursing service may improve if a nurse’s empathy is high. However, to date, there are insufficient studies on the role of nurses’ empathy in the relationship between caring behavior and the quality of nursing services.

Therefore, this study examines the relationship between nurses’ caring behavior, quality of nursing services, and empathy. Moreover, this study explores the mediating effect of empathic competence on the relationship between caring behavior and quality of nursing services, and thus provides basic data to develop a program to improve the quality of nursing services.

## 2. Materials and Methods

### 2.1. Design 

This study explores the mediating effect of compassion competence on the relationship between caring behavior and quality of nursing services. 

### 2.2. Participants

The participants were nurses working for more than three months in patient nursing as their main job at South Korean university hospitals. Only those who understood the purpose and necessity of the study and voluntarily agreed to participate were included in this study. The number of participants required for multiple regression analysis was calculated using the G*power 3.1 program. Considering the effect size as 0.15 (moderate), significance level (α) of 0.05, power of 0.95, and 11 general characteristics, the number of participants required, with caring behavior and compassion as independent variables and quality of nursing services as the dependent variable, was calculated as 189. However, considering the fidelity of the survey results, 220 questionnaires were collected, of which 9 were excluded due to insufficient responses; finally, 211 were used for the analysis.

### 2.3. Measurements

The questionnaire consisted of 72 items: 11 items regarding general characteristics and career, 24 items evaluating caring behavior, 20 items measuring the quality of nursing services, and 17 items evaluating compassion competence.

#### 2.3.1. Caring Behavior

For caring behavior, a tool developed by Wolf et al. [18], modified by Wu et al. [19], and translated into Korean by Kim and Kwon [20] was adopted. This tool consists of 24 caring behavior inventory (CBI) items: 8 for confidence, 5 for professional support and skill, 6 for respect, and 5 for bonding. It is measured on a 6-point Likert scale, and higher scores indicate a higher frequency of caring behavior. In the study by Wu et al. [20], the reliability of the tool was Cronbach’s α = 0.96, and in this study, it was 0.93.

#### 2.3.2. Quality of Nursing Services

A tool developed by Parasuraman et al. [21] and translated and revised by Lee [22] was used to assess the quality of nursing services. It consists of 20 items in the 5 categories of: tangible, reliable, responsible, secure, and empathetic. These are measured on a 5-point Likert scale; higher scores indicate higher quality of nursing services, as perceived by nurses. The reliability of the tool in Lee’s study [22] was Cronbach’s ɑ = 0.94; in this study, Cronbach’s α = 0.92.

#### 2.3.3. Compassion Competence

The compassion competence measurement tool developed by Lee and Seomun [23] was used. This tool consists of 17 items measured on a 5-point Likert scale. The higher the score, the higher the nurse’s empathy. In Lee and Seomun’s study [23], the reliability of the tool was Cronbach’s α = 0.93, and in this study, it was 0.90.

### 2.4. Data Collection

Data for this study were collected from 1 November to 31 December 2020. Prior to data collection, we visited the nursing department of two university hospitals located in city D, a nearby population, and explained the necessity and purpose of the study as well as the contents of the questionnaire to the head of the nursing department. We then obtained their consent to proceed with the questionnaire. Then, questionnaires were distributed to the head nurses in the wards. Only those nurses who voluntarily agreed to the survey after understanding the research purpose and necessity from the head nurse were selected as participants. They responded to the survey using a self-reported questionnaire after filling out a written consent form and were provided with a predetermined case. 

### 2.5. Data Analysis

The collected data were analyzed using the SPSS/WIN 24.0 program. Descriptive statistics were used for participants’ general characteristics. Pearson’s correlation coefficients were used to identify the correlation between caring behavior, quality of nursing services, and compassion competence. Before performing Pearson’s correlation coefficient analysis, normality, homoscedasticity, and independence were checked. The normality of the data was evaluated using the skewness and kurtosis, and homoscedasticity was evaluated using Bartlett’s sphericity test. In addition, independence was evaluated using Durbin-Watson’s test, and it was confirmed that all were satisfied. The mediating effect of compassion competence on the relationship between caring behavior and quality of nursing services was analyzed through hierarchical multiple regression. The significance test of the mediating effect of compassion competence on the relationship between caring behavior and quality of nursing service was analyzed by the Sobel test. Reliability was measured through Cronbach’s α.

## 3. Results

### 3.1. Differences in Caring Behavior, Quality of Nursing Services, and Compassion Competence According to General Characteristics

The participants’ general characteristics and differences in caring behavior, quality of nursing services, and compassion competency according to general characteristics are presented in Table 1. The average age of the participants was 31.8 years (±6.54), with those in their 30s or younger accounting for 50.2% of the sample. Among the participants, 69.2% had a bachelor’s degree, whereas 16.1% had an associate’s degree; 40.3% were married, and 36.5% reported belonging to a religion. The average nurse’s career was 8.34 (±5.58) years, and 44.1% were general ward nurses. Staff nurses constituted 90.5% of the sample, and 70.2% worked in shifts. Overall, 46.9% reported being satisfied with their job and only 12.3% said they were satisfied with their salary. The mean score for caring behavior was 3.65 (±0.59); for the quality of nursing services, 3.73 (±0.46); and for compassion competence, 3.82 (±0.43). 

Caring behavior was correlated with the presence of a spouse (t = −2.38, *p* = 0.018), position (t = 4.51, *p* = 0.012), job satisfaction (F = 11.43, *p* < 0.001), and salary satisfaction (F = 4.30, *p* = 0.015). Caring behavior was higher in those with a spouse than those without, and it was higher in head nurses than in general nurses. Moreover, caring behavior was higher among those with high job satisfaction. Those with average salary satisfaction showed higher caring behavior than those who were dissatisfied.

Quality of nursing services was correlated with religion (t = −2.08, *p* = 0.039), job satisfaction (F = 14.31, *p* < 0.001), and salary satisfaction (F = 3.52, *p* = 0.031). The quality of nursing services was higher among those who were religious, and who had higher satisfaction with their job and salary.

Differences in compassion competence were correlated with religion (t = −2.24, *p* = 027), work type (t = 2.23, *p* = 0.027), job satisfaction (F = 14.33, *p* < 0.002), and salary satisfaction (F = 5.94, *p* = 0.003). Religious people and those who worked fixed hours had higher compassion competence. Moreover, those who were satisfied with their jobs and salaries had higher compassion competence than those who were dissatisfied.

### 3.2. Correlation between Study Variables

Caring behavior was positively correlated with the quality of nursing services (r = 0.59, *p* < 0.001) and compassion competence (r = 0.61, *p* < 0.001). Additionally, there was a positive correlation between quality of nursing services and compassion competence (r = 0.73, *p* < *0*.001) (Table 2).

### 3.3. Mediating Effects of Compassion Competence on the Relationship between Caring Behavior and Quality of Nursing Services

We calculated the Durbin-Watson value to verify the independence of the residuals before testing the mediating effect; the value was found to be within 1.81–2.00, which was close to 2, indicating that the dependent variable had no autocorrelation. To check for multicollinearity, we calculated the tolerance limit, which was 0.62, all below 1.0, and the variance expansion coefficient for all variables, which ranged from 1.00–1.61, all less than 10. Thus, there was no problem of multicollinearity. This finding confirmed that the model was suitable for regression analysis.

The results of the three-step regression analysis to verify the mediating effect of compassion competence are shown in Table 3. As a result of the regression analysis of step 1, we confirmed that the independent variable, caring behavior, had a statistically significant effect on compassion competence (β = 0.61, *p* < 0.001). In step two of the regression analysis, the influence of the independent variable, caring behavior, on the dependent variable, the quality of nursing services, was confirmed. This result was also confirmed to be statistically significant (β = 0.59, *p* < 0.001). In step three, to analyze the influence of compassion competence as a parameter on the quality of nursing services as a dependent variable, regression analysis was performed with caring behavior and compassion competence as independent variables and quality of nursing services as the dependent variable. Both caring behavior (β = 0.23, *p* = 0.007) and compassion competence (β = 0.58, *p* < 0.001) impacted the quality of nursing services. If both the independent variables and the parameter are significant in step three and the coefficient of the independent variable in step three is smaller than that of the independent variable in step two, it can be said that the parameter has a partial mediating effect on the relationship between the independent and the dependent variables. In other words, the independent variable has a direct influence on the dependent variable, and the independent variable has an indirect influence on the dependent variable by changing the parameter. The regression analysis revealed that both caring behavior and compassion competence were significant in step three, and the coefficient of caring behavior in step three was lesser than the coefficient of caring behavior in step two. Compassion competence was found to have a partial mediating effect, and the explanatory power was 56.6%. We conducted the Sobel test to verify the significance of compassion competence, and it was confirmed as a partial parameter (Z = 8.03, *p* < 0.001) in the relationship between caring behavior and quality of nursing services.

## 4. Discussion

This study provides fundamental data for the development of a program to improve the quality of nursing services by identifying the level of compassion competence, quality of nursing services, and caring behaviors of nurses, and by confirming the mediating effect of compassion competence in the relationship between caring behavior and nursing service quality.

The level of caring behavior of participants in this study was scored at 3.65, which is lower than that reflected in Kim and Choi’s study [24] targeting intensive care unit (ICU) nurses and Eo and Kim’s study [25] of long-term care hospital nurses. This may be due to the difference between the study participants of the previous studies [24,25] and the nursing participants of this study. Due to the characteristics of the hospital environment in Korea, the level of holistic care is higher in the ICU than in the general ward. In addition, the bond between patients and nurses can be strengthened because one nurse takes care of several patients in the general ward and a small number of patients in the ICU. Similarly, nursing hospitals may exhibit higher levels of care compared to general wards, as most patients find it difficult to take care of themselves due to old age or dementia. It was found that the caring behavior of the participants of this study differed according to marital status, position, and job and salary satisfaction. This result is similar to that of Eo and Kim’s study [25] on differences in caring behavior according to marital status and position. In this study, there was no difference in nursing behavior according to the age of the nurses; however, in the studies conducted by Kim and Choi [24] and Eo and Kim [25], older nurses demonstrated higher levels of caring behavior. It was difficult to locate previous studies that measured the difference in caring behavior according to job satisfaction or salary satisfaction. In the future, programs implemented to improve nurses’ caring behavior should consider education reflecting the characteristics of the nurses.

The quality of nursing service score was 3.73, and there was a significant difference according to religion, job satisfaction, and salary satisfaction. The studies conducted by Lee [22] and Yeom and Seo [26] reported scores of 3.77 and 3.69, respectively using the same tool. In this study, there was no difference in nursing service quality scores according to general characteristics, whereas in several previous studies [4,26] differences in the nursing service quality scores were found according to general characteristics such as education, educational background, and position. This can be due to the differences in target hospitals, as nursing service quality differs according to hospital size and nursing grade [26]. Moreover, unlike previous studies, this study used variables related to work environment satisfaction, and all these variables were found to affect the quality of nursing services. A recent study on the nurse’s working environment showed that satisfaction with the working environment leads to positive outcomes such as peace of mind or compassion for nurses [27,28]. Thus, to improve the quality of nursing services, it is necessary to establish a systematic education program according to the nurses’ individual characteristics and explore their working environment from various perspectives.

In this study, the compassion competence score was 3.82, which was relatively higher than that in Lee and Seomun [23] targeting nurses at tertiary hospitals. The compassion competence of the participants of this study differed according to religion, work type, job satisfaction, and salary satisfaction. In contrast, in a previous study, there were significant differences according to age, education level, and clinical experience or position [29]. Although the age and educational level of the participants of this study were similar to those of a previous study [29], they showed different results. Therefore, it is necessary to systematically examine the general characteristics that affect empathy. Contrary to the belief that nurses’ empathy differed according to individual characteristics, this study confirmed that variables related to their work environment, such as work type, job satisfaction, and salary satisfaction, were significantly related to nurses’ empathy. However, since the participants of this study were limited to nurses in some hospitals, repeated studies with more nurses are needed in the future.

This study found that nurses’ compassion competence had a significant positive correlation with caring behavior and the quality of nursing services. Additionally, compassion competence was found to have a partial mediating effect on the relationship between caring behavior and quality of nursing services. In other words, compassion can be improved by performing caring actions, which can, in turn, enhance the quality of nursing services. This implies that the compassion competence of nurses is important to improve the quality of nursing services during nurses’ caring behavior. Nurses provide care during interaction with patients by actively identifying patient needs based on professional knowledge and skills [11,12]. To sensitively discover patient needs, nurses must take a deep interest in them, and demonstrate the skill to observe meticulously, and the attitude to respond directly to the patients’ needs and actively solve them [15]. The nurses’ compassion can be a source of providing individualized care to patients. In addition, patient care is expressed through the relationship between the nurse and the patient rather than through the one-sided nursing provision [11,12]. By understanding the patients’ physical, spiritual, and emotional difficulties, nurses can express understanding and compassion toward patients and provide them with encouragement and emotional support [30,31]. Such empathic relationship-centered nursing improves the quality of care [32]. Therefore, nurses will be able to gradually improve their caring behavior by developing empathy and understanding toward patients. Therefore, providing high-quality care allows nurses to build therapeutic and meaningful relationships with patients, and enhance patient satisfaction [33].

The limitation of this study is its small sample size, which causes issues with generalizability. Therefore, repeated studies with large samples are needed in the future. In addition, since this study used a self-report questionnaire, possible acquiescence tendencies or social desirability biases should not be overlooked. In the future, it is necessary to develop a tool that can objectively measure the compassion competence of nurses and the quality of nursing services from the patient’s point of view. However, the significance of this study is that it is meaningful as it explores the relationship between caring behavior, quality of nursing services, and compassion competence among nurses. The results of this study can be used as fundamental data for developing programs to improve the quality of nursing services in the future.

## 5. Conclusions

This study tried to provide fundamental data highlighting strategies to improve the quality of nursing services by examining the mediating effect of nurses’ compassion competence on the relationship between caring behavior and the quality of nursing services. It was revealed that there was a positive correlation between caring behavior, quality of nursing services, and compassion competence. In addition, it was confirmed that the compassion competence of nurses had a partial mediating effect on the relationship between caring behavior and the quality of nursing services. On this basis, we propose the following: first, it is necessary to include factors for improving nurses’ compassion competence in educational programs designed to improve the quality of nursing services from a clinical point of view; second, education for nursing students to have the correct knowledge and awareness of caring behavior and compassion competence should be provided at the same time to improve compassion competence; and third, iterative research is needed to expand the scope of the survey, and in-depth research is needed to compare the differences in compassion competence and perception of nursing service quality between nurses and participants.

## Figures and Tables

**Table 1:** Differences in caring behavior, quality of nursing services, and compassion competence according to general characteristics (*N* = 211).

Characteristics	Categories	M ± SD or n (%)	Caring Behavior			Quality of Nursing Service			Compassion Competence		
			M ± SD	t or F	*p* Scheffe	M ± SD	t or F	*p* Scheffe	M ± SD	t or F	*p* Scheffe
Gender	Male	4 (1.9)	3.78 ± 0.28	0.87	0.437	3.54 ± 0.36	1.07	0.359	3.60 ± 0.42	−1.06	0.363
	Female	207 (98.1)	3.65 ± 0.60			3.74 ± 0.46			3.83 ± 0.43		
Age (yr)		31.80 ± 6.54									
	≤30	106 (50.2)	3.55 ± 0.57	2.98	0.053	3.73 ± 0.49	2.74	0.066	3.76 ± 0.45	2.95	0.054
	31~39	83 (39.3)	3.73 ± 0.56			3.69 ± 0.39			3.87 ± 0.38		
	≥40	22 (10.4)	3.81 ± 0.74			3.94 ± 0.50			3.97 ± 0.48		
Educational level	Associate’s degree	34 (16.1)	3.74 ± 0.79	0.97	0.380	3.71 ± 0.61	1.27	0.281	3.87 ± 0.55	1.55	0.215
	Bachelor’s degree	146 (69.2)	3.61 ± 0.56			3.71 ± 0.44			3.79 ± 0.41		
	Master’s degree	31 (14.7)	3.73 ± 0.45			3.86 ± 0.28			3.93 ± 0.38		
Marital status	Yes	85 (40.3)	3.77 ± 0.61	−2.38	0.018	3.75 ± 0.44	−0.45	0.649	3.89 ± 0.41	−1.83	0.068
	No	126 (59.7)	3.57 ± 0.57			3.72 ± 0.47			3.78 ± 0.44		
Belonging to a religion	Yes	77 (36.5)	3.71 ± 0.68	−1.04	0.299	3.82 ± 0.48	−2.08	0.039	3.91 ± 0.47	−2.24	0.027
	No	134 (63.5)	3.62 ± 0.53			3.68 ± 0.44			3.77 ± 0.40		
Position	Staff nurse ^a^	191 (90.5)	3.62 ± 0.59	4.51	0.012 a < c	3.73 ± 0.46	0.18	0.834	3.81 ± 0.43	1.05	0.352
	Charge nurse ^b^	15 (7.1)	3.81 ± 0.51			3.78 ± 0.38			3.87 ± 0.34		
	Head nurse ^c^	5 (2.4)	4.36 ± 0.45			3.82 ± 0.47			4.09 ± 0.66		
Unit	General ward	93 (44.1)	3.63 ± 0.61	0.54	0.705	3.71 ± 0.42	0.50	0.729	3.78 ± 0.43	1.02	0.396
	Intensive care unit	49 (23.2)	3.67 ± 0.50			3.75 ± 0.43			3.88 ± 0.43		
	Emergency room	9 (4.2)	3.45 ± 0.49			3.64 ± 0.36			3.66 ± 0.28		
	Operation room	21 (10.0)	3.62 ± 0.68			3.71 ± 0.57			3.82 ± 0.52		
	Others	39 (18.5)	3.74 ± 0.63			3.81 ± 0.53			3.90 ± 0.40		
Career (yr)		8.34 ± 5.58									
	≤3	46 (21.8)	3.49 ± 0.66	2.70	0.069	3.65 ± 0.52	1.09	0.338	3.77 ± 0.50	1.22	0.297
	4~10	99 (46.9)	3.65 ± 0.57			3.75 ± 0.43			3.80 ± 0.42		
	≥11	66 (31.3)	3.76 ± 0.55			3.77 ± 0.44			3.89 ± 0.40		
Rotation	Fixed	63 (29.9)	3.77 ± 0.62	1.89	0.061	3.81 ± 0.50	1.56	0.120	3.93 ± 0.43	2.23	0.027
	Rotation	148 (70.1)	3.60 ± 0.57			3.70 ± 0.43			3.78 ± 0.42		
Job satisfaction	Satisfaction ^a^	99 (46.9)	3.82 ± 0.58	11.43	<0.001 a > b > c	3.90 ± 0.43	14.31	<0.001 a > b > c	3.96 ± 0.41	14.33	<0.001 a > b > c
	Usual ^b^	65 (30.8)	3.61 ± 0.59			3.63 ± 0.46			3.78 ± 0.39		
	Dissatisfaction ^c^	47 (22.3)	3.34 ± 0.48			3.53 ± 0.38			3.58 ± 0.42		
Salary satisfaction	Satisfaction ^a^	26 (12.3)	3.76 ± 0.62	4.31	0.015 b > c	3.91 ± 0.46	3.52	0.031 a > b > c	4.04 ± 0.41	5.94	0.003 a > c
	Usual ^b^	66 (31.3)	3.79 ± 0.64			3.78 ± 0.51			3.88 ± 0.42		
	Dissatisfaction ^c^	119 (56.4)	3.54 ± 0.54			3.67 ± 0.41			3.74 ± 0.43		

Values with superscript letters a, b and c are significantly different across rows (*p* < 0.05).

**Table 2:** Correlations among study variables (*N* = 211).

	1	2	3
	r (*p*)	r (*p*)	r (*p*)
1. Caring behavior	1		
2. Quality of nursing service	0.59 (<0.001)	1	
3. Compassion competence	0.61 (<0.001)	0.73 (<0.001)	1

**Table 3:** Mediating effect of compassion competence on the relationship between caring behavior and quality of nursing services (*N* = 211).

Step	Independent Variables	Dependent Variables	B	SE	Β	t (*p*)	Adj. R^2^	F (*p*)
1	Caring behavior	Compassion competence	0.47	0.04	0.61	11.30 (<0.001)	0.376	127.71 (<0.001)
2	Caring behavior	Quality of nursing service	0.43	0.04	0.59	10.78 (<0.001)	0.354	116.31 (<0.001)
3	Caring behavior	Quality of nursing service	0.17	0.04	0.23	4.11 (<0.001)	0.566	138.17 (<0.001)
	Compassion competence	Quality of nursing service	0.55	0.05	0.58	10.15 (<0.001)		
			Sobel test; Z = 8.03 (*p* < 0.001)					

Adj. = adjusted; SE = standard error.

## Data Availability

Not applicable.
